# Possible Interaction Between Physical Exercise and Leptin and Ghrelin Changes Following Roux-en-Y Gastric Bypass in Sarcopenic Obesity Patients—A Pilot Study

**DOI:** 10.3390/nu16223913

**Published:** 2024-11-15

**Authors:** Cláudia Mendes, Manuel Carvalho, Jorge Bravo, Sandra Martins, Armando Raimundo

**Affiliations:** 1 Unidade Local Saúde Alentejo Central, EPE—Hospital Espírito Santo de Évora, 7000-811 Évora, Portugal; 2 CRI.COM—Centro Responsabilidade Integrada de Cirurgia da Obesidade e Metabólica, 7000-811 Évora, Portugal; 3 CHRC—Comprehensive Health Research Centre, Universidade de Évora, 7004-516 Évora, Portugal; 4 Departamento de Desporto e Saúde, Escola de Saúde e Desenvolvimento Humano, Universidade de Évora, 7004-516 Évora, Portugal; 5 CBIOS—Research Center for Biosciences & Health Technologie, Universidade Lusófona, 1749-024 Lisboa, Portugal; 6 Research Center in Sports Sciences, Health and Human Development (CIDESD), 5000-801 Vila Real, Portugal; 7 Departamento de Desporto, Universidade Europeia, 1500-210 Lisboa, Portugal

**Keywords:** exercise, bariatric surgery, leptin, ghrelin, sarcopenia, sarcopenic obesity

## Abstract

Introduction: Leptin and ghrelin are two hormones that play a role in weight homeostasis. Leptin, which is produced primarily by adipocytes and is dependent on body fat mass, suppresses appetite and increases energy expenditure. Conversely, ghrelin is the “hunger hormone”, it stimulates appetite and promotes fat storage. Bariatric surgery significantly alters the levels and activity of these hormones, contributing to weight loss and metabolic improvements. Clarifying the interplay between bariatric surgery, weight loss, physical exercise, leptin, and ghrelin is essential in developing comprehensive strategies for optimizing the long-term outcomes for candidates who have undergone bariatric surgery, especially for sarcopenic patients. Methods: This was a randomized controlled study with two groups (n = 22). The patients in both groups had obesity and sarcopenia. A Roux-en-Y-gastric bypass (RYGB) procedure was performed on all patients. The intervention group participated in a structured exercise program three times per week, beginning one month after surgery and lasting 16 weeks. Patient assessment was performed before surgery (baseline) and after the completion of the exercise program. The control group received the usual standard of care and was assessed similarly. Results: After surgery, weight, BMI, and lean mass decreased significantly in both groups between the baseline and the second assessment. Leptin levels were not significantly different between baseline and the second assessment in the physical exercise group, but were significantly lower in the control group (*p* = 0.05). Ghrelin levels increased over time in both groups, but the differences were not significant. When we associated leptin (the dependent variable) with weight (the independent variable), we found that lower weight was associated with lower leptin levels. A similar relationship was also observed between the leptin and sarcopenia parameters (muscle strength and mass), as well as in the bone health parameters (bone mineral density and t-score). Higher ghrelin levels were significantly associated with higher t-scores and z-scores (*p* < 0.05). Conclusion: Exercise has been shown to have a significant effect on leptin and ghrelin levels after bariatric surgery. By incorporating regular physical activity into their lifestyle, bariatric patients can optimize their weight loss outcomes and improve their overall health. After the physical exercise protocol, patients in the intervention group revealed more established leptin levels, which may indicate a protected pattern concerning decreased leptin levels. An unfavorable profile was evidenced, according to which greater weight loss, sarcopenia, and osteoporosis were associated with lower leptin levels.

## 1. Introduction

Obesity has become a significant public health challenge worldwide, with its prevalence rising steadily over the past few decades. The World Health Organization has highlighted obesity as a global health concern, affecting millions of individuals struggling to maintain a healthy weight [1,2].

Bariatric surgery is the most effective therapeutic approach for achieving significant and sustained weight loss in individuals with severe obesity. The surgical procedure facilitates weight loss and induces profound metabolic changes, which improve or resolve obesity-associated conditions [3].

The prevalence of sarcopenia conditions in patients with obesity varies between 10% and 50% [4]. Post-bariatric surgery patients with sarcopenic obesity face significant clinical challenges. Preoperative sarcopenia has been proven to be a good predictor of perioperative complications and death after major abdominal surgeries and, in older people, the risk of cardiovascular events in the perioperative period increases [5,6].

Despite the undeniable benefits of bariatric surgery, understanding the underlying mechanisms that contribute to its success remains an active research area. The impact of this process on the complex hormonal regulation of appetite and metabolism is not fully understood. Hormonal alterations after surgery are of particular interest [7,8].

Two such hormones, leptin and ghrelin, may play crucial roles in regulating energy, appetite balance, and body weight, and their levels can be significantly altered following bariatric surgery [8]. Leptin, which is produced by adipose tissue, acts on the hypothalamus to suppress appetite and increase energy expenditure [9]. However, following bariatric surgery, significant weight loss often leads to decreased leptin levels, which can have profound implications for appetite regulation and metabolic function. This can lead to increased hunger and decreased energy expenditure, making it challenging to maintain weight loss [10].

Ghrelin, which is predominantly secreted by the stomach, has the opposite effect, stimulating appetite and promoting food intake. Alterations in leptin and ghrelin levels have been observed in individuals with obesity and after bariatric surgery, highlighting their importance in the physiological response to weight loss interventions [10,11].

Regular exercise is known to improve cardiovascular health, enhance metabolic function, and promote psychological well-being [12,13]. In the context of bariatric surgery, exercise is recommended as a complementary intervention to maximize weight loss, maintain muscle mass, and improve overall health outcomes. The interplay between exercise and hormonal changes after surgery, particularly concerning leptin and ghrelin, is an area that has garnered increasing scientific interest [14].

Several studies have demonstrated the positive impact of exercise on hormone regulation after bariatric surgery [15]. Regular exercise could play an important role in enhancing leptin sensitivity; improving appetite control and metabolic function; and, after bariatric surgery, in contributing to the promotion of long-term weight maintenance [10]. In some studies, exercise has been found to decrease ghrelin secretion and to suppress appetite, leading to better control over food intake. By incorporating regular exercise into their routine, individuals who have undergone bariatric surgery could eventually better manage their ghrelin levels and reduce their cravings for high-calorie foods [16].

Therefore, exercise has been shown to increase leptin sensitivity, decrease ghrelin secretion, and improve overall metabolic function [17]. By engaging in regular exercise routines that include both aerobic and resistance training components, individuals may enhance their hormonal balance after surgery and increase sustainable weight loss efforts [18].

However, the specific impact of exercise on leptin and ghrelin levels in individuals after bariatric surgery remains an area of active research. Understanding how exercise influences these hormonal changes can provide valuable insights into optimizing weight loss outcomes and preventing weight regain in bariatric patients.

Several mechanisms have been proposed to explain the potential effects of exercise on leptin and ghrelin regulation [19]. Exercise-induced changes in body composition, such as increased muscle mass and reduced fat mass, may alter leptin sensitivity and secretion. Additionally, acute and chronic exercise modulate appetite-regulating hormones, including ghrelin, in both lean and obese individuals. These physiological adaptations may contribute to the success of exercise interventions in promoting weight loss and long-term weight maintenance after bariatric surgery [20].

This study explored the effects of a regular exercise program on leptin and ghrelin levels in patients with sarcopenic obesity, following bariatric surgery.

## 2. Methods

### 2.1. Study Design

This study was part of the EXPOBAR protocol, NCT0528921 [21], which is ongoing in a single center for metabolic and bariatric surgery in Portugal [22,23].

Recruitment took place between December 2021 and December 2023 from among candidates who met the diagnostic criteria for bariatric surgery. Patients were randomized into a control group (CG) or an intervention group (IG) by either a bariatric surgeon or a sports specialist nurse. The data were collected from the hospital’s electronic patient records.

The participants who agreed to participate in the study read and confirmed the free and informed consent form, which had been previously approved by the University and Hospital Ethics Committee (HESE_CE_1917/21).

**Figure 1:**
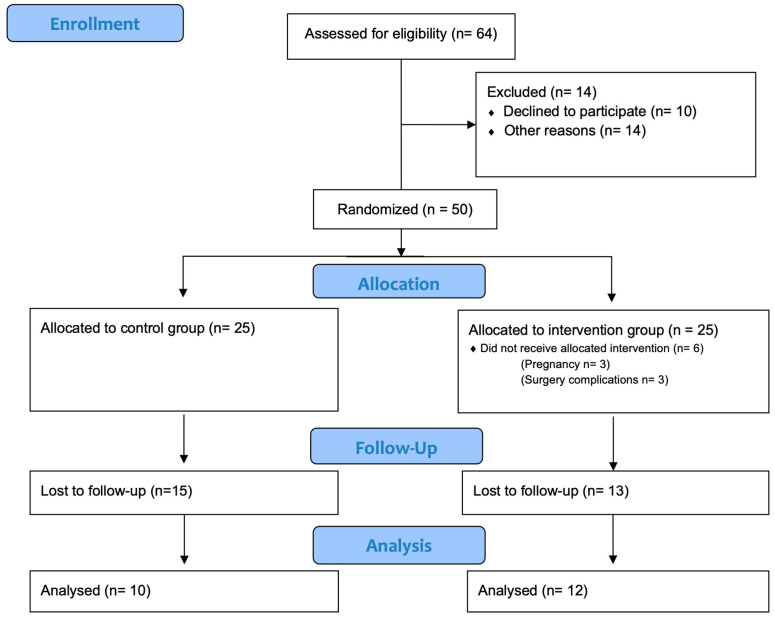
Consort flow diagram [24].

Exercise training began one month after surgery, with a frequency of three times per week, up to a maximum of 55 min per session, for 16 weeks. This study included two evaluations: before surgery and after exercise training. All assessments were conducted by researchers who were blinded to the study’s objectives and the participants’ group allocation to minimize potential biases and ensure the integrity of the data collected. This study protocol complied with the CONSORT 2010 recommendations (Figure 1) (Supplementary Materials).

### 2.2. Eligibility Criteria

The eligibility criteria included a body mass index (BMI) ≥ 40 kg/m^2^ or BMI ≥ 35 kg/m^2^, at least one obesity-related comorbidity, aged between 18 years and 60 years, a diagnosis of sarcopenic obesity based on the EASO/ESPEN criteria, no contraindication to exercise practice, and having agreed to participate in the study.

The exclusion criteria were patients with problems in locomotion, other previous bariatric surgery, and RYGB complications during surgery.

### 2.3. Sample Size and Randomization

This study was a secondary analysis of the registered randomized controlled trial, NCT0528921, at Clinicaltrials.gov [21]. G*Power software version 3.1 was utilized for sample size determination [25]. A total of 22 participants were selected to identify a moderate estimated effect size of at least 0.99 standard deviations for between-group differences in the sarcopenia outcome risk [26,27]. ANOVA repeated measures were conducted, focusing on within–between interactions, with an alpha level set at α = 0.05 and statistical power at 1 − β = 0.75. This study was a non-blinded RCT, which included a 16-week combined exercise intervention, alongside a control group receiving standard care.

Patients who had undergone bariatric surgery (gastric bypass/RYGB) were randomly assigned either to usual care (CG) or to usual care combined with an exercise program (IG) using a computer-generated randomization system, this was completed by the surgeon at the proposal stage. Both groups underwent follow-up sessions with a psychologist and a nutritionist.

### 2.4. Intervention

A progressive combined exercise program involves both aerobic and strength training. The exercise prescription was based on the FITT-VP principles (frequency, type, intensity, time, type, duration, volume, and progression) for people with obesity [28,29,30].

**Figure 2:**
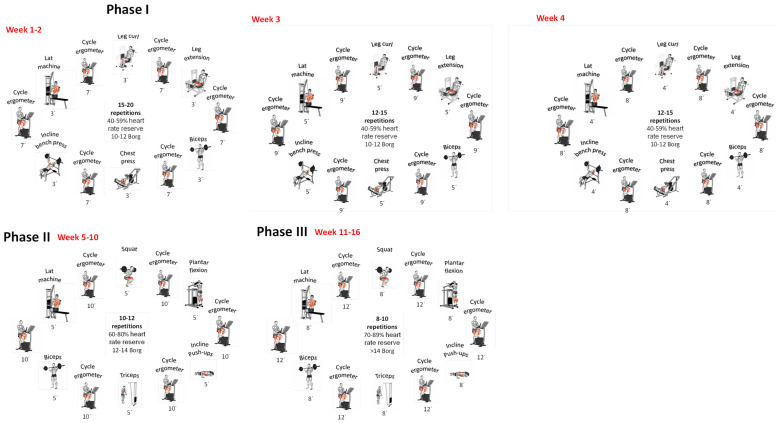
Exercise training periodization.

The program was to be completed in 16 weeks, three times a week, for 55 min per session, starting a month after surgery, following our previous work [31,32] and recommendations from the World Health Organization (WHO) (5) and the American College of Sports Medicine (ACSM) [29] (Figure 2).

The participants in the IG completed the 16-week exercise training program. Each session was composed of 5 min of specific warm-ups on a treadmill (a); (b) resistance training (weeks 1–4); (c) hypertrophy training (weeks 5–10); (d) strength training (weeks 11–16); and a 10-min flexibility cool-down (myofascial release, mobility, static stretching, and dynamic stretching).

Interval training and circuit strength training methods were included in the first phase. The phases in the central block were increased by 10 min, followed by an assessment of the patient’s response. To assess the perceived effort of the exercise performed, heart rate reserve and the Borg scale values were recorded following a continuous progression [33].

### 2.5. Outcomes

The details of the intervention have been described previously [21]. Two assessment moments were performed, the first before surgery and the second after the exercise program. The CG participants were instructed to maintain their current activities. Data collection was carried out in two stages: baseline assessment and after 16 weeks of intervention.

Anthropometry and body composition: Weight (kg) and height (cm) were measured to calculate BMI (kg/m^2^). Body composition was assessed via dual-energy X-ray absorptiometry (DEXA) (DXA, Hologic QDR, Hologic, Inc., Bedford, MA, USA). During this procedure, the participants were asked to fast and to be without metal items or adornments. The total weight loss percentage (%TWL) was also calculated based on the initial weight and actual weight (weight loss/initial weight (Kg/Kg)).

Perioperative blood samples—leptin and ghrelin: Blood sampling was performed before surgery and after the exercise program’s completion. The fasting blood samples were collected and processed immediately according to the hospital protocol. The results were assessed one week later at the hospital database.

**Figure 3:**
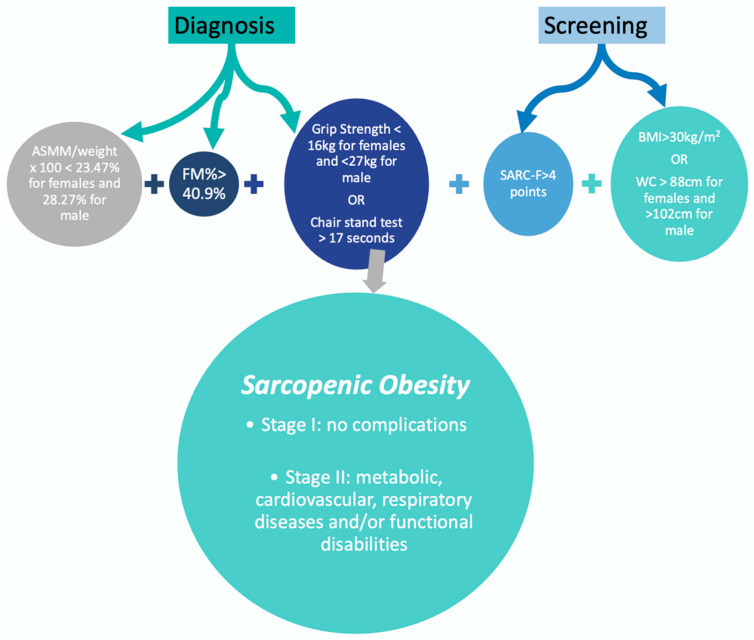
Algorithm of sarcopenic obesity diagnostic [36].

Sarcopenia: Sarcopenic obesity was defined as a high BMI or waist circumference, combined with low muscle mass and low muscle strength [34,35,36]. Low muscle strength was defined by handgrip strength for reduced strength (cut-offs < 27 Kg for M and <16 Kg for F) and low muscle mass by DEXA, based on ASMM/weight × 100 (cut-offs < 28.27% for M and <23.47% for F) (Figure 3).

### 2.6. Statistical Methods

Statistical analyses were conducted using JAMOVI version 2.3.19. Descriptive statistics were expressed as the mean ± standard deviation (SD) for parametric data and as the median ± standard deviation (SD). Data normality was assessed with the Shapiro—Wilk test. Two-way ANOVA was used to compare the dependent variables, considering group and two-time points before and after the exercise program. Cohen’s effect size was also calculated for the interaction of treatments. Relevance was interpreted as small (*d* = 0.2), medium (*d* = 0.5), or large (*d* = 0.8) [37]. Linear and logistic regression analyses were performed to analyze correlations between variables’ significance levels, which were set at *p* < 0.05.

## 3. Results

A total of 22 patients with sarcopenic obesity were randomized: 12 were assigned to the IG and 10 were assigned to the CG. All patients in the IG completed the intended intervention. The baseline characteristics of the sample are summarized in Table 1. The baseline characteristics of both groups were similar, although there was a trend towards a difference in weight in the IG (*p* = 0.067).

Significant weight loss was observed in both groups (Table 2). The intervention group experienced a smaller weight reduction (*d* = 0.425).

Physical function, as evaluated by a handgrip dynamometer, decreased significantly in the CG. In the IG, the difference was not significant. For the sarcopenic parameter, the impact of the exercise intervention was significant (*p* = 0.050; *d* = 0.500). Similar results were obtained for BMC, with a significant impact of the exercise being observed (*p* = 0.004; *d* = 0.733).

**Figure 4:**
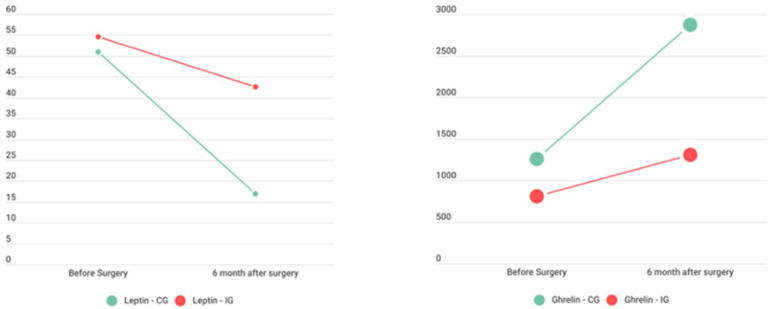
Leptin and ghrelin evaluation after surgery and post-proposed exercise program.

After the RYGB, ghrelin levels increased, but their difference from the baseline levels reached significance after 6 months. The results for the fasting ghrelin concentrations were different between the groups but not significant at the final time point. The fasting leptin levels decreased significantly at six months in the CG but not in the IG, which is a potential indicator of exercise benefits. This difference was statistically significant between the two groups (Figure 4).

Associations between the leptin and ghrelin levels and the clinical variables related to the sarcopenic obesity parameters were examined via multivariable linear regression. We found that weight (*r* = 0.475; *p* = 0.009); BMI (*r* = 0.625; *p* = 0.022); bone mineral density (*r* = 0.709; *p* = 0.011); muscle mass and strength (*r* = 0.689; *p* = 0.014); and *t*-scores (*r* = 0.510; *p* = 0.045) were positively associated with the leptin levels. The *t*-scores (CG: *r* = 0.578, *p* = 0.040; IG: *r* = 0.640, *p* = 0.012) and *z*-scores (CG: *r* = 0.673, *p* = 0.016; IG: *r* = 0.628, *p* = 0.014) were positively correlated with the ghrelin levels (Table 3).

## 4. Discussion

This study aimed to explore the impact of exercise on the regulation of the leptin and ghrelin levels in individuals with sarcopenic obesity, who had recently undergone bariatric surgery. This was the first randomized control trial to evaluate and relate the evolution of leptin and ghrelin levels after bariatric surgery in patients with sarcopenic obesity. Our results showed that exercise directly influences leptin sensitivity and can contribute to stabilizing ghrelin levels, suggesting a promising complementary approach to optimizing post-operative outcomes.

We anticipate that this study’s findings will provide novel insights into how exercise modulates leptin and ghrelin levels in patients who have undergone bariatric surgery. Specifically, we expect improvements in leptin sensitivity and reduced ghrelin levels due to exercise interventions [10]. These hormonal changes will likely enhance appetite control, increase energy expenditure, and improve weight loss maintenance [38].

Previous studies have already shown that the significant weight loss induced by bariatric surgery results in a reduction in leptin levels, an expected effect due to the decrease in fat mass, which is the main producer of this hormone [39,40,41]. This reduction is expected and correlates with weight loss. However, the role of exercise in modulating leptin levels after bariatric surgery has had different interpretations [8]. In this study, leptin levels decreased significantly in the CG. In the IG, it was firmly established that weight loss positively improves leptin sensitivity and that exercise plays a crucial role in regulating this mechanism. The stabilization of leptin levels in the intervention group suggests that exercise plays a protective role, preventing abrupt drops in leptin, which could make it difficult to maintain weight loss and could affect appetite control in the long term. Min et al. revealed that, 2 years after bariatric surgery, greater weight loss was associated with a greater reduction in leptin, but there was no effect on adiponectin levels after 4 years of follow-up [42,43].

However, several studies have demonstrated that exercise can influence leptin levels independently of weight loss [42,43]. Exercise is known to improve leptin sensitivity, which can be diminished in individuals with obesity due to leptin resistance. This improvement in leptin sensitivity means that the body can respond more effectively to hormones, potentially enhancing appetite regulation and energy balance. The results of this research have helped to elucidate how exercise impacts leptin levels and sensitivity in post-bariatric surgery patients, which is essential in developing comprehensive postoperative care strategies that optimize long-term weight loss and metabolic health.

Unlike leptin, ghrelin levels increase before meals and decrease afterward, reflecting its role in meal initiation [39,44]. Bariatric surgery—particularly procedures that involve significant anatomical changes to the stomach, such as sleeve gastrectomies and RYGBs—drastically alters ghrelin production.

The impact of RYGBs on ghrelin concentrations has been widely studied, with controversial results [44]. Some groups have reported a significant decrease in ghrelin levels after RYGBPs [44,45,46]. These low levels of ghrelin after RYGBs could account for increased satiety and reduced food intake, helping to explain the long-term effects this surgery has on patients with severe obesity. However, other studies have not reported changes in ghrelin after RYGBs, suggesting that they are unlikely to contribute to suppressing food intake in the postoperative stage [47,48]. In accordance with the results of this study, some studies have reported higher ghrelin concentrations after an RYGBP than before surgery [49,50,51].

In addition, the association between higher ghrelin levels and higher BMD and t- and z-scores in our study raises important questions about the role of this hormone in preserving bone health after bariatric surgery. The existing literature has shown controversial results regarding changes in ghrelin levels after bariatric surgery, with some studies reporting a decrease in levels and others, like ours, showing an increase. This variability can be explained by methodological differences among the studies, including the type of surgery, the length of follow-up, and the characteristics of the population studied.

Physical exercise, especially resistance training and combined aerobics, has been shown to have important effects on hormone regulation, which included profound effects on ghrelin levels. Acute bouts of exercise generally reduce ghrelin concentrations, which may help suppress appetite postexercise. In our study, the intervention group showed an attenuation in the increase in ghrelin compared to the control group. Although the increase in ghrelin was expected due to the body’s adaptation to weight loss, the fact that the intervention group showed a less pronounced increase suggests that exercise can modulate ghrelin secretion and help to control appetite. Regular exercise may, therefore, play an important role in preventing weight regain after surgery, contributing to appetite control and improving satiety in the long term [9,52]. Regular exercise training, however, has a more nuanced adaptation concerning ghrelin regulation [52], which can vary depending on the intensity and duration of the exercise regimen. Investigating how different types and intensities of exercise influence ghrelin levels in the context of bariatric surgery is crucial for understanding the hormonal adaptations that support weight loss and maintenance.

The leptin produced in adipocytes seems to be related to sarcopenic obesity, as it can reduce the capacity of myocytes for protein synthesis [53]; however, the evidence concerning muscle strength is inconsistent [54]. In this study, the results revealed that a decrease in leptin was associated with a reduction in muscle strength. This may suggest that patients who have already been diagnosed with obesity-related sarcopenia and who, after bariatric surgery, have a more significant decrease in muscle strength, have a greater decrease in leptin levels and, consequently, in satiety levels. This may make them prone to weight gain in the long term.

The effects of leptin on bone mass and the regulation of bone metabolism are also unclear [55,56]. The current results indicate that a greater decrease in leptin levels is associated with a significant reduction in bone mineral content, although this effect was less pronounced in the exercise group. This demonstrated the protective effect of exercise, with a minimal impact on the t-score and z-score. This allowed us to corroborate the results reported by Mohammadi et al., that leptin can be an important biomarker for diagnosing osteoporosis [39].

The protective effects of exercise observed in our results also extended to preserving muscle strength and lean mass. The association between decreased leptin and reduced muscle strength in the control group suggested that excessive loss of leptin may be related to the loss of muscle mass, particularly in sarcopenic patients. These findings reinforced the importance of including a post-surgical exercise program to prevent muscle deterioration and to maintain physical functionality. Similarly, the preservation of bone mineral density in the intervention group indicated that physical exercise may be a protective factor against osteoporosis in patients undergoing bariatric surgery, as suggested in other studies.

Finally, our results offer a promising insight into the role of exercise in hormonal modulation after bariatric surgery. The positive impact of exercise on leptin and ghrelin optimizes weight loss. It can improve patients’ quality of life by helping to control appetite, maintain muscle mass, and preserve bone health. These findings reinforce the need to routinely include structured exercise programs into the postoperative care of bariatric patients, especially those with sarcopenic obesity.

This study had limitations, including its small sample size and the limited duration of the follow-up. Future research ought to incorporate a larger sample size and an extended intervention period to evaluate the sustained effects of exercise on hormone levels, as well as their relationship with the maintenance of weight loss and long-term metabolic health. Furthermore, investigating various types and intensities of exercise may yield further insights into the most effective strategies for optimizing post-surgical hormone regulation.

## 5. Conclusions

Bariatric surgery represents a powerful tool in the fight against obesity, offering significant and sustained weight loss for individuals with severe obesity. However, the success of this intervention depends on a comprehensive approach, which includes lifestyle modifications such as exercise. The hormonal adaptations induced by exercise, particularly leptin and ghrelin, may be critical in understanding and optimizing postoperative outcomes.

Exercise has been shown to have a significant effect on leptin and ghrelin levels after bariatric surgery. By incorporating regular physical activity into their lifestyle, bariatric patients can optimize their weight loss outcomes and improve their overall health. Further research is needed to fully understand the mechanisms by which exercise influences hormone regulation post-surgery, but current evidence suggests that physical exercise could be key to long-term success for bariatric patients.

## Figures and Tables

**Table 1:** Baseline characteristics.

Parameter (Mean ± SD)	Intervention Group n = 12	Control Group n = 10	*p* Value
Sex (% female)	75%	90%	0.388
Age (years)	44.08 ± 13.2	50.4 ± 11.1	0.240
Weight (kg)	117.1 ± 15.8	103.6 ± 16.9	0.067
BMI (kg/m^2^)	43.1 ± 5.2	41.8 ± 3.4	0.388
Leptin (ng/mL)	54.6 ± 29.8	50.9 ± 28.5	0.355
Ghrelin (pg/mL)	811 ± 763	1261 ± 1424	0.773

BMI—body mass index.

**Table 2:** Main outcomes.

	Baseline		6 Months		*Sig.*	*d*
	CG	IG	CG	IG		
Weight (kg)	103.55 ± 16.86	117.08 ± 15.79	73.5 ± 13.2 ^a^	83.0 ± 12.4 ^a^	*p* = 0.099	0.425
BMI (kg/m^2^)	41.8 ± 3.40	43.10 ± 5.17	29.4 ± 2.62 ^a^	30.6 ± 4.37 ^a^	*p* = 0.821	0.067
Leptin (ng/mL)	50.9 ± 28.47	54.6 ± 29.75	17.0 ± 18.0 ^a^	42.5 ± 44.1	*p* = 0.050	0.013
Ghrelin (pg/mL)	1261 ± 1424	811 ± 762.72	2870 ± 2230	1311 ± 968	*p* = 0.175	0.067
Body fat (%)	46.60 ± 3.23	46.7 ± 6.47	39.5 ± 5.91 ^a^	37.2 ± 8.02 ^a^	*p* = 0.107	0.417
Handgrip (kg)	20.60 ± 7.18	25.5 ± 6.87	16.4 ± 5.79 ^a^	22.2 ± 7.09	*p* = 0.050	0.500
Lean mass (kg)	53.45 ± 12.48	58.19 ± 8.02	45.23 ± 11.47 ^a^	43.38 ± 9.07 ^a^	*p* = 0.456	0.200
BMC (kg)	2.33 ± 0.44	2.50 ± 0.37	1.96 ± 0.17 ^a^	2.42 ± 0.37	*p* = 0.004	0.733
BMD (g/cm^2^)	1.14 ± 0.13	1.21 ± 0.17	1.10 ± 0.08	1.16 ± 0.12	*p* = 0.276	0.283
Total body t-score	0.43 ± 1.51	0.54 ± 1.51	−0.07 ± 0.68	0.76 ± 1.23	*p* = 0.306	0.267
Total body z-score	0.55 ± 1.14	0.49 ± 1.22	0.15 ± 0.46	0.49 ± 1.23	*p* = 0.842	0.058

BMI—body mass index; BMD—bone mineral density; BMC—bone mineral content. ^a^ Post hoc significance between evaluations, *p* < 0.001; *d* = Cohen effect size; and *Sig.* = group effect.

**Table 3:** Analysis of variables and leptin and ghrelin levels after exercise.

	Leptin (ng/mL)				Ghrelin (pg/mL)			
	CG		IG		CG		IG	
	*r*	*p* Value	*r*	*p* Value	*r*	*p* Value	*r*	*p* Value
%TWL (%)	−0.518	0.937	0.194	0.273	0.353	0.159	0.314	0.160
Weight (kg)	0.475	0.009	0.102	0.376	0.356	0.156	−0.145	0.673
BMI (kg/m^2^)	0.625	0.022	0.051	0.431	0.137	0.353	−0.167	0.716
Body fat (%)	0.359	0.154	0.225	0.241	0.230	0.205	0.040	0.245
Handgrip (kg)	0.689	0.014	−0.068	0.658	0.027	0.470	−0.097	0.618
Lean mass (kg)	0.718	0.010	−0.316	0.841	0.502	0.028	−0.151	0.680
BMC (g)	0.144	0.304	−0.094	0.561	0.348	0.162	−0.084	0.612
BMD (g/cm^2^)	0.709	0.011	−0.008	0.510	0.341	0.167	0.208	0.258
Total body t-score	0.171	0.319	0.510	0.045	0.578	0.040	0.640	0.012
Total body z-score	0.197	0.293	0.283	0.186	0.673	0.016	0.628	0.014

BMI—body mass index; BMD—bone mineral density; BMC—bone mineral content; TWL—total weight loss. *r*—Pearson coefficient; Significance defined as *p* < 0.05.

## Data Availability

The data presented in this study are available upon request from the corresponding author. The data are not available publicly due to ethical, legal, or commercial restrictions.

## Supplementary Materials

The following supporting information can be downloaded at: https://www.mdpi.com/article/10.3390/nu16223913/s1. CONSORT 2010 checklist of information to include when reporting a randomised trial.
